# Recurrence Risk of Liver Cancer Post-hepatectomy Using Machine Learning and Study of Correlation With Immune Infiltration

**DOI:** 10.3389/fgene.2021.733654

**Published:** 2021-12-08

**Authors:** Xiaowen Qian, Huilin Zheng, Ke Xue, Zheng Chen, Zhenhua Hu, Lei Zhang, Jian Wan

**Affiliations:** ^1^ Department of Information and Electronic Engineering, Zhejiang University of Science and Technology, Hangzhou, China; ^2^ Department of Biological and Chemical Engineering, Zhejiang University of Science and Technology, Hangzhou, China; ^3^ Division of Hepatobiliary and Pancreatic Surgery, Department of Surgery, Fourth Affiliated Hospital, School of Medicine, Zhejiang University, Yiwu, China; ^4^ Key Laboratory of Combined Multi-Organ Transplantation, Division of Hepatobiliary and Pancreatic Surgery, Department of Surgery, First Affiliated Hospital, School of Medicine, Zhejiang University, Ministry of Public Health Key Laboratory of Organ Transplantation, Hangzhou, China; ^5^ Division of Hepatobiliary and Pancreatic Surgery, Yiwu Central Hospital, Yiwu, China

**Keywords:** liver cancer, recurrence risk, machine learning, immune infiltration, TCGA

## Abstract

Postoperative recurrence of liver cancer is the main obstacle to improving the survival rate of patients with liver cancer. We established an mRNA-based model to predict the risk of recurrence after hepatectomy for liver cancer and explored the relationship between immune infiltration and the risk of recurrence after hepatectomy for liver cancer. We performed a series of bioinformatics analyses on the gene expression profiles of patients with liver cancer, and selected 18 mRNAs as biomarkers for predicting the risk of recurrence of liver cancer using a machine learning method. At the same time, we evaluated the immune infiltration of the samples and conducted a joint analysis of the recurrence risk of liver cancer and found that B cell, B cell naive, T cell CD4^+^ memory resting, and T cell CD4^+^ were significantly correlated with the risk of postoperative recurrence of liver cancer. These results are helpful for early detection, intervention, and the individualized treatment of patients with liver cancer after surgical resection, and help to reveal the potential mechanism of liver cancer recurrence.

## Introduction

Liver cancer is a common malignancy with morbidity and mortality ranking sixth and fourth, respectively. (Bray et al., 2018). Surgical resection is the most common treatment method for liver cancer. However, the high recurrence and metastasis rate of liver cancer patients after resection poses a great challenge to liver cancer treatment. According to statistics, the recurrence rate of liver cancer patients 3 years after surgery is approximately 40–50%, and the recurrence rate 5 years after surgery is as high as 60–70%. (Nakayama and Takayama, 2014). Therefore, it is of great clinical significance to identify high-risk patients with recurrence of liver cancer after radical surgical resection.

With the development of high-throughput sequencing technology, some molecular biomarkers have been reported to predict liver cancer recurrence after hepatectomy. In previous studies, Erb-B2 receptor tyrosine kinase 2 (*ERBB2)* and NUF2 component of the NDC80 Kinetochore Complex (*NUF2)* were reported to be biomarkers of hepatocellular carcinoma (HCC) recurrence after surgery. (Li et al., 2017a), (Feng et al., 2021) D. Wang et al. (Chen et al., 2020) demonstrated that the level of interleukin 11 (*IL11*) increased after hepatectomy, which led to the growth of HCC. Zhou et al. (Chen et al., 2021) confirmed that WNK lysine deficient protein kinase 2 (*WNK2)* is a driving factor of HCC and a risk factor for early recurrence through genome sequencing. However, due to the complex etiology of liver cancer (such as hepatitis virus infection, alcohol-related liver disease, nonalcoholic fatty liver) and the difficulty of collecting liver cancer samples with a history of recurrence after hepatectomy, the predictive effect of individual genes screened from a limited number of samples in previous studies may be limited and may not be generally applicable to liver cancer caused by different etiologies. Our study included 306 liver cancer samples caused by different etiologies from The Cancer Genome Atlas, which is the largest sample size that can be incorporated with both recurrent disease history and sequencing data currently, improving the reliability of prediction.

Machine learning is a mathematical method for finding patterns in data to achieve artificial intelligence, and is now widely used in medical image detection and medical aid diagnosis. (Li et al., 2017a; Chen et al., 2020; Chen et al., 2021; Feng et al., 2021). Some studies have used machine learning methods to predict the recurrence of liver cancer. Ho et al.(Ho et al., 2006) constructed a KNN model based on 18 patients to predict HCC recurrence after resection. Liang et al. (Liang et al., 2014) analyzed 83 patients with HCC after radio frequency ablation and constructed an SVM model to predict HCC recurrence. However, it is worth noting that KNN and SVM have good predictive performance, but they are commonly used to solve classification problems and are not very explanatory for variables. Random forest in machine learning is an ensemble classifier, and the tree-based ensemble makes it suitable for handling with redundant features. (Reif et al., 2006). Unlike KNN, SVM and random forest, logistic regression is a regression analysis that gives formulaic results to quantify the probability of event occurrence, with good interpretation of variables. (Nick and Campbell, 2007).

In our study, we aimed to identify potential biomarkers of liver cancer recurrence after resection, and construct a model to quantify the risk of recurrence based on the biomarkers. The above advantages of logistic regression make it a suitable algorithm for our study. In order to solve the problem that logistic regression is difficult to handle with high-dimensional variables, we performed random forest to screen variables. The model constructed using this combined method allows for the quantification of the risk of liver cancer recurrence and good predictive performance.

## Materials and Methods

### Patient Selection

The RNA-seq data and clinical data of samples with liver cancer were downloaded from The Cancer Genome Atlas database (https://portal.gdc.cancer.gov/). The detailed clinical data included age, sex, TNM stage, histologic grade, and recurrence (Supplementary Table S1). Each file downloaded was the mRNA expression data of one sample, and we collated all sample data into one file to obtain an mRNA expression matrix of all samples, while matching the clinical information to the corresponding samples. The inclusion criteria for the cohort were as follows: 1) Normal tissue samples were removed. 2) Samples with R0 excision were selected for further analyses. Next, according to whether new tumor events occurred after the initial treatment, the patients were divided into recurrence and non-recurrence groups. Patients who had a new tumor event after the initial treatment and the type of new tumor event were intrahepatic recurrence, locoregional recurrence, or extrahepatic recurrence were included in the recurrence group. Samples that did not develop new tumor events after the initial treatment were included in the non-recurrence group. Finally, 306 usable samples were obtained, including 158 non-recurrent and 148 recurrent samples. The clinical characteristics of the recurrence and non-recurrence groups are shown in Supplementary Table S2.

In the subsequent analysis, the samples were further divided into two subgroups based on etiology: the alcohol-associated liver disease subgroup (ALD, n = 57) and the hepatitis virus infection-related subgroup (HVI, n = 98). The HVI subgroup included those associated with hepatitis B virus (HBV, n = 71) and hepatitis C virus (HCV, n = 27) infections. Considering the small sample size of HCV, HCV and HBV were not split into two subgroups and were uniformly classified as the HVI subgroup. The remaining patients (n = 151) were not included in the subgroup analysis due to missing etiologies.

### Screening of DE-mRNAs

DESeq and EdgeR packages in R software (https://www.r-project.org/) were used to screen DE-mRNAs between the recurrence and non-recurrence groups. The threshold was set at *p* < 0.05, and |log2 (fold change)|>1. Overlapping DE-mRNAs screened using the two packages were used for further analyses.

### Functional Enrichment and Co-expression Network

Gene Ontology (GO) functional enrichment of these DE-mRNAs was conducted using the database for annotation, visualization, and integrated discovery (https://david.ncifcrf.gov/), and *p* < 0.05, was set as the cutoff value. The co-expression network between DE-mRNAs was predicted using the STRING database (https://string-db.org/) and visualized using Cytoscape (https://cytoscape.org/).

### Estimation of Immune Cells and Identification of Differential Immune Cells

Based on the mRNA expression levels of the recurrence and non-recurrence groups, we used the TIMER database (http://timer.cistrome.org/) to estimate the expression of immune cells in the two groups.

### Development of the Risk Assessment Model

The overall flow chart of this study was shown in Supplementary Figure S1. 306 patients were assigned to the training cohort (n = 215) or validation cohort (n = 91) with a 7:3 split ratio by applying simple random sampling. In the training cohort, 60 DE-mRNAs were entered into random forest (RF) as variables. RF is an ensemble method based on multiple decision trees, and the decision trees in RF are built by randomly selected samples made from bootstrap and a randomly selected subset of variables. Some original samples were not selected to construct trees, which are called out-of-bag (OOB) dataset. (Breiman, 2001). After inputting 60 variables into RF, mean decrease accuracy (MDA) and mean decrease Gini (MDG) provided in RF were calculated to assess the importance scores of each variable for liver cancer recurrence. MDA quantifies the importance of a variable by calculating the mean decrease accuracy in the OOB before and after the permutation of the variable, and MDG quantifies the importance of a variable by measuring the mean decrease in Gini impurity caused by the variable when it is used to form a split in the random forest. (Díaz-Uriarte and de Andrés, 2006; Wang et al., 2016). These were achieved by the “randomForest” package in R software. Next, a variable ranking was obtained by ranking the importance scores. Larger values of the importance scores represented greater influence of DE-mRNAs on recurrence. The variable ranking can be described as:

$$X_{Rank}=\left(x^{r\left(1\right)},x^{r\left(2\right)},\cdots ,x^{r\left(d\right)}\right)$$

where  r(i),i=1,2,⋯,d is the index of variable $x_{i}$ in the descending ranking. We took MDA as the importance scores to obtain the top 30 importance ranking of variables ${\tilde{X}}_{Rank1}$ , and took MDG as the importance scores to obtain the top 30 importance ranking of variables ${\tilde{X}}_{Rank2}$ . In order to obtain a small scale of variables and increase reliability, we took the intersection of the variables in ${\tilde{X}}_{Rank1}$ and ${\tilde{X}}_{Rank2}$ as the variables to be put into the risk assessment model, which can be presented as:

$$X={\tilde{X}}_{Rank1}\cap {\tilde{X}}_{Rank2}$$

After the above steps, 18 DE-mRNAs with important effects on liver cancer recurrence were obtained as the input variables for the risk assessment model.

Multivariate logistic regression was used to construct the risk assessment model for liver cancer recurrence. The calculation formula of risk score can be presented as:

$$Risk\;score=\frac{1}{1+{\text{e}}^{-\left(\omega^{T}X+b\right)}}$$

In our study, X is the expression level of each DE-mRNA, and the parameters ω and b can be learned from the training data. The parameter ω can be interpreted as the relative impact of each DE-mRNA in the recurrence of liver cancer. In the training cohort, 18 DE-mRNAs screened by random forest were entered into logistic regression, and the model was validated using the validation cohort.

In addition, a logistic regression model without variable screening (model 2), a logistic model with stepwise regression (model 3), and a logistic model with L1 regularization (model 4) were constructed for comparison. In model 2, a logistic regression model was constructed directly with 60 DE-mRNAs. Model 3 was constructed by stepwise logistic regression with Akaike information criterion (AIC) for feature selection, which is a classic variable selection method. (Agostini et al., 2015; Sanchez-pinto et al., 2018; Hu et al., 2019).Model 4 added the L1 regularization term to the logistic regression, which can have a dimensionality reduction effect. (Tibshirani, 1996; Wang et al., 2014). The “glmnet” package in R software was used for the analyses. StromalScore, ImmuneScore, and ESTIMATEScore are three scores used to assess the level of infiltrating stromal and immune cells. (Yoshihara et al., 2013). They were calculated to compare with the risk score presented in our study, using the “estimate” package in R software.

### Performance Evaluation of the Prediction Models

The receiver operating characteristics (ROC) curve and the area under the ROC curve (AUC) were used to evaluate the performance of the models. As measures of model performance, they exhibit some desirable properties and are good ways to visualize model performance. (Bradley, 1997). In the field of big biological data and cancer-related research, ROC and AUC are widely used to evaluate the performance of machine learning models. (Le et al., 2020; Yu et al., 2020; Kudo et al., 2021; Le et al., 2021). ROC and AUC analyses were performed by R software.

### Statistical Analysis

All statistical analyses were performed using GraphPad Prism version 8.0 software (GraphPad Software Inc.). A two-tailed *t*-test was used to identify immune cells that were significantly different between the recurrence and non-recurrence groups. In all analyses, a two-tailed *p*-value less than 0.05, was considered statistically significant. Random forest and logistic regression models were performed using R software (version 4.0.2). The codes for this study have been uploaded on GitHub (https://github.com/polarbbbear/code).

## Results

### Identification of DE-mRNAs and the Link Between DE-mRNAs

**FIGURE 1:**
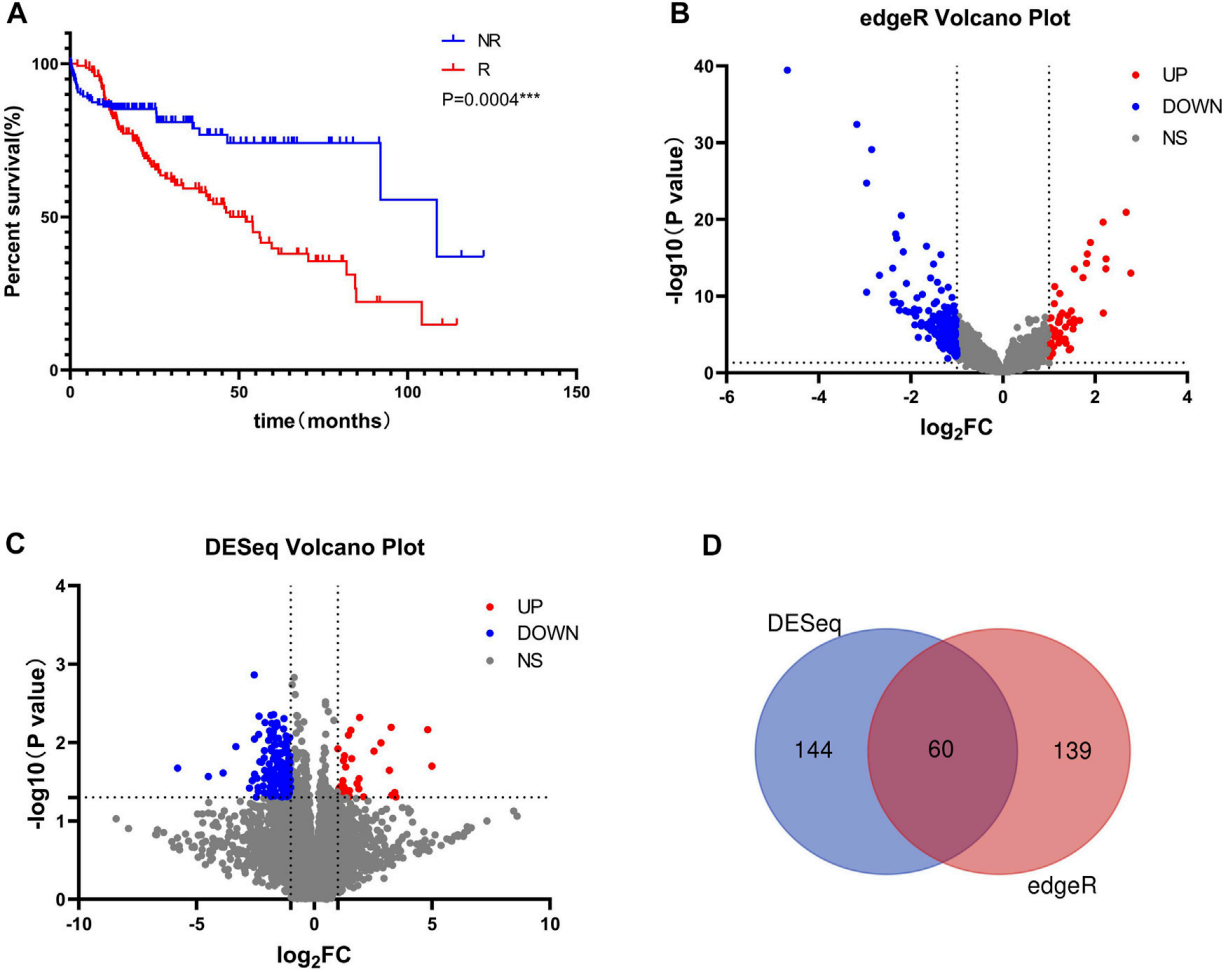
Identification of DE-mRNAs **(A)** Kaplan-Meier curves of OS between the recurrence and non-recurrence groups across the entire dataset **(B)** DE-mRNAs that identified using the edgeR package **(C)** DE-mRNAs that identified using the DESeq package **(D)** Overlapping DE-mRNAs between the selection methods.

The clinical information of the entire data, training data, and validation data are presented in Supplementary Table S1. Kaplan-Meier curves indicated a significant difference in prognosis between the recurrence and non-recurrence groups (Figure 1A). Using the edgeR and DESeq packages of R, 199 and 204 mRNAs that were significantly different between the recurrence and non-recurrence groups were identified, respectively (Figures 1B, C). Next, 60 overlapping DE-mRNAs were obtained through the cross between the differentially expressed mRNAs identified by the edgeR package and DESeq package (Figure 1D). To unveil the relationships among all DE-mRNAs, we constructed a network diagram of protein interactions by the string database (Supplementary Figure S2A). In order of the ability to interact with the others, the top 10 genes were CEA Cell Adhesion Molecule 5 (*CEACAM5*), Mucin 1, Cell Surface Associated (*MUC1*), Cathepsin G (*CTSG*), Ret Proto-Oncogene (*RET*), CD79a Molecule (*CD79A*), Collagen Type XI Alpha 2 Chain (*COL11A2*), GLI Family Zinc Finger 2 (*GLI2*), Collagen Type X Alpha 1 Chain (*COL10A1*), Myosin Light Chain 3 (*MYL3*), Tectorin Beta (*TECTB*). Interestingly, we found that most of these key node genes play important roles in tumorigenesis (*RET*, *CEACAM5*), immune regulation (*CTSG*, *CD79A*) and the EMT pathway (*COL10A1*, *COL11A2*), suggesting their significant role in the recurrence of liver cancer. To quantify the interaction relationships between DE-mRNAs, we calculated the correlation of DE-mRNAs and found that many genes of the immunoglobulin superfamily were highly positively correlated (Supplementary Figure S2B), such as genes of the Immunoglobulin Heavy Variable (IGHV) and Immunoglobulin Kappa Variable (IGKV) regions. The combination of these genes may co-regulate immune function in patients and affect the recurrence of liver cancer after surgery.

### Functional Enrichment for mRNAs Co-expressed

**FIGURE 2:**
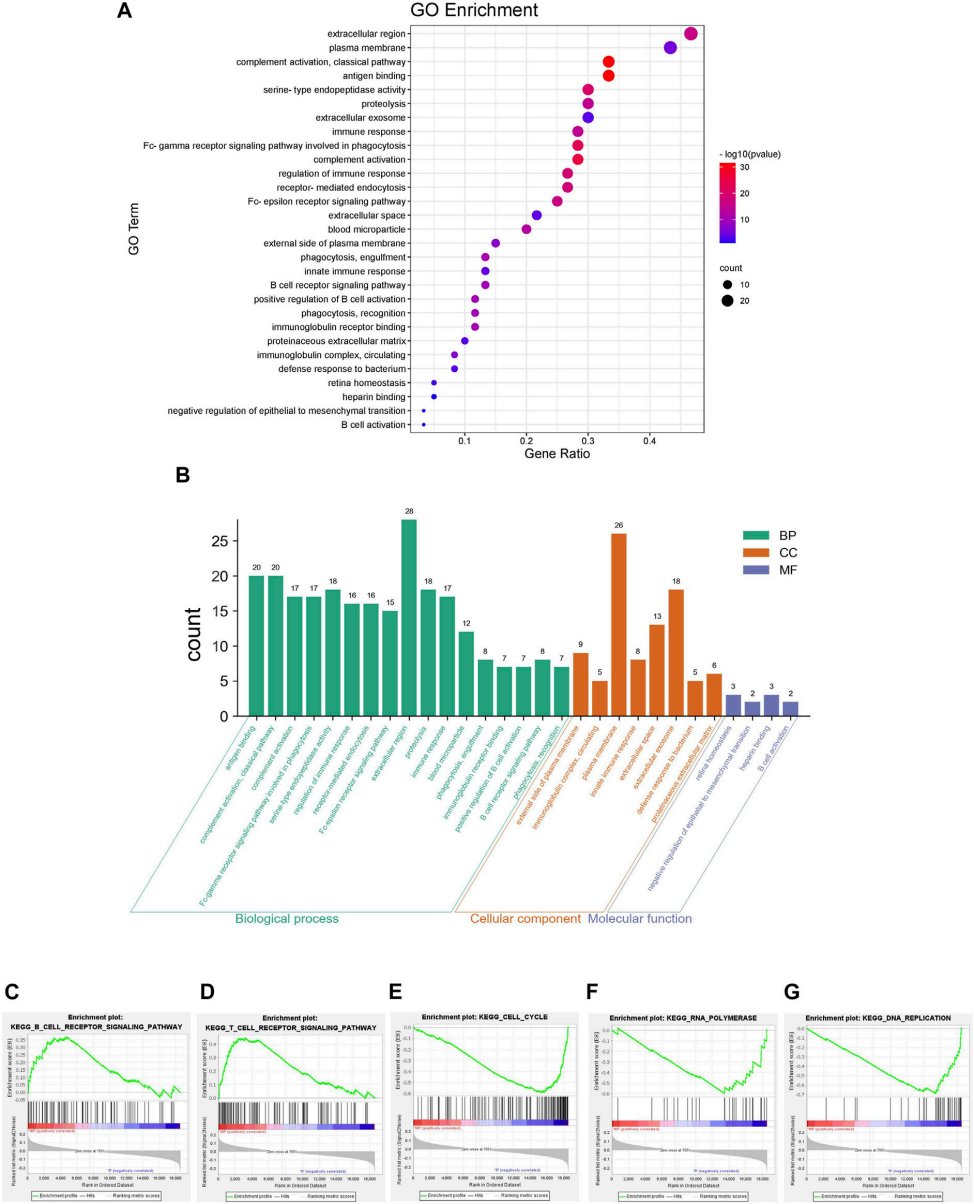
Functional enrichment for mRNAs co-expressed **(A)** The bubble pattern shows the enrichment pathways with Gene Ratio, gene count and *p*-value **(B)** The histogram shows the enrichment of molecular function, biological process and cellular component. Results of GSEA enrichment on **(C)** B cell receptor signaling pathway **(D)** T cell receptor signaling pathway **(E)** Cell cycle **(F)** RNA polymerase, and **(G)** DNA replication.

To comprehensively study the potential mechanism of liver cancer recurrence, functional enrichment was performed in both groups. The results of the GO enrichment analysis are shown in Figures 2A, B. It was noted that the DE-mRNAs between the two groups were significantly enriched in immune-related pathways such as antigen binding, Fc-gamma receptor signaling pathway involved in phagocytosis, regulation of immune response, immune response, immunoglobulin receptor binding, positive regulation of B cell activation, B cell receptor signaling pathway, immunoglobulin complex and circulating, innate immune response, and B cell activation. Moreover, GSEA enrichment results showed that there were significant differences in the B cell receptor signaling pathway, T cell receptor signaling pathway, cell cycle, RNA polymerase, and DNA replication between the recurrence and non-recurrence groups. The B cell receptor signaling pathway and the T cell receptor signaling pathway-related genes were significantly enriched in the non-recurrence group, while the cell cycle, RNA polymerase, and DNA replication pathway-related genes were significantly enriched in the recurrence group (Figures 2C–G). These results suggested that the changes of immune-related functions may be the mechanism influencing liver cancer recurrence.

### Identification of Immune Cells With Significant Difference Between Two Groups

**FIGURE 3:**
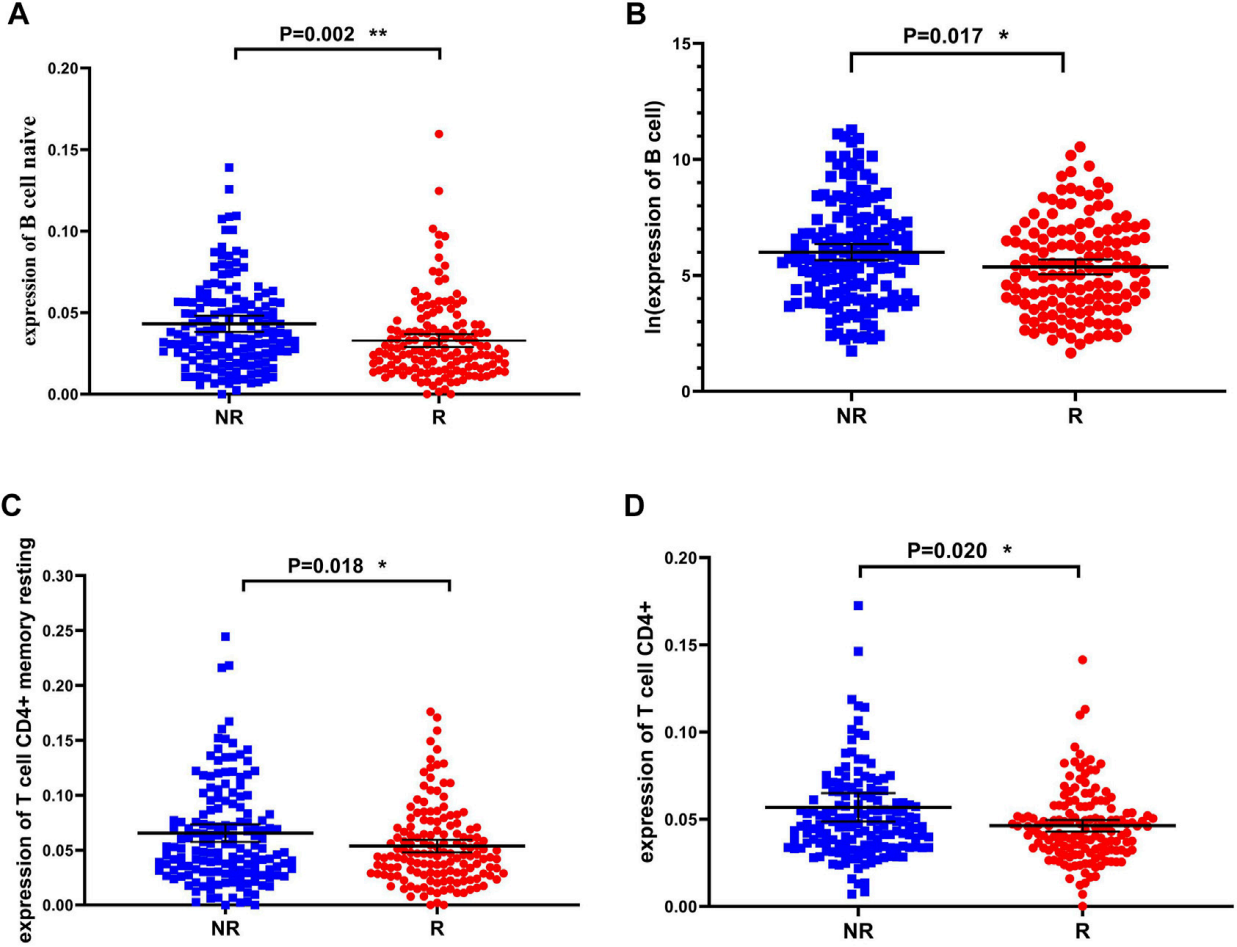
Immune cells that are significantly different between the recurrence and non-recurrence groups **(A)** B cell naive expression between the two groups **(B)** B cell expression between the two groups **(C)** T cell CD4^+^ memory resting expression between the two groups **(D)** T cell CD4^+^ expression between the two groups.*p < 0.05, **p < 0.01 and ***p < 0.001.

The results of functional enrichment suggested that there were significant differences in immune-related pathways between the recurrence and non-recurrence groups. In order to investigate which immune cells are involved in the mechanisms influencing the recurrence of liver cancer, we used the TIMER database (http://timer.cistrome.org) to estimate the expression level of immune cells in the two groups, and the *t*-test was used to determine whether there were significant differences in the expression of immune cells between the two groups. Immune cells showed significant differences between the recurrence and non-recurrence groups (Figures 3A–D), and the expression of naive B cells, B cells, T cell CD4^+^ memory resting, and T cell CD4^+^ were downregulated in the recurrence group. The results demonstrated the significant influence of these four immune cells on liver cancer recurrence.

### Construction and Performance Evaluation of the Risk Assessment Model

**FIGURE 4:**
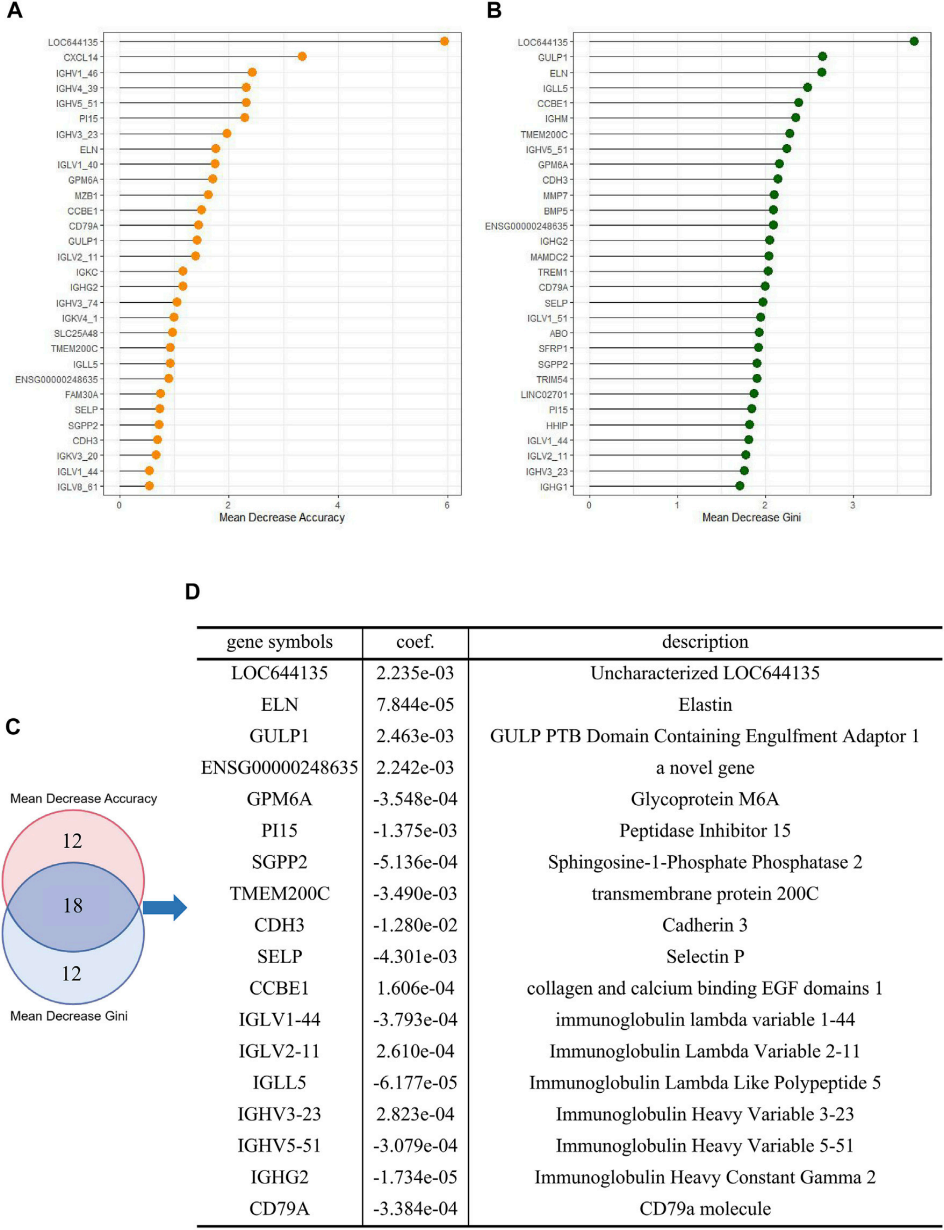
Construction of a risk assessment model **(A)** Top 30 DE-mRNAs with mean decrease Gini **(B)** Top 30 DE-mRNAs with mean decrease accuracy **(C)** Overlapping DE-mRNAs between the selection methods **(D)** Logistic risk model constructed by overlapping DE-mRNAs.

To identify the mRNAs that play an important role in liver cancer recurrence, we constructed 500 decision trees using random forest with 60 DE-mRNAs as features and measured the importance of each DE-mRNA on recurrence by calculating the mean decrease Gini and mean decrease accuracy for each DE-mRNA. The top 30 DE-mRNAs ranked by mean decrease Gini and mean decrease accuracy were selected (Figures 4A, B). The top 30 mRNAs were intersected, and 18 overlapping mRNAs were finally selected to construct the risk assessment model (Figures 4C, D).

Subsequently, we trained an 18-mRNA risk assessment model in the training cohort using logistic regression analysis. These 18 mRNAs were uncharacterized LOC644135 (*LOC644135),* elastin (*ELN),* GULP PTB domain-containing engulfment adaptor 1 (*GULP1)*, *ENSG00*000248635 (a novel gene), Glycoprotein M6A (GPM6A), peptidase inhibitor 15 (PI15), Sphingosine-1-Phosphate phosphatase 2 (SGPP2), transmembrane protein 200C (TMEM200C), cadherin 3 (CDH3), selectin P (SELP), collagen and calcium binding EGF domains 1 (CCBE1), immunoglobulin lambda variable 1–44 (IGLV1-44), Immunoglobulin Lambda Variable 2–11 (IGLV2-11), Immunoglobulin Lambda Like Polypeptide 5 (IGLL5), Immunoglobulin Heavy Variable 3–23 (IGHV3-23), Immunoglobulin Heavy Variable 5–51 (IGHV5-51), Immunoglobulin Heavy Constant Gamma 2 (IGHG2), and CD79a molecule (CD79A). The risk assessment model was developed based on the coefficients of mRNAs and the constant derived from this analysis. The value of the constant b in the logistic regression formula was 0.3883, and the coefficients of mRNAs were shown in Figure 4D.

**FIGURE 5:**
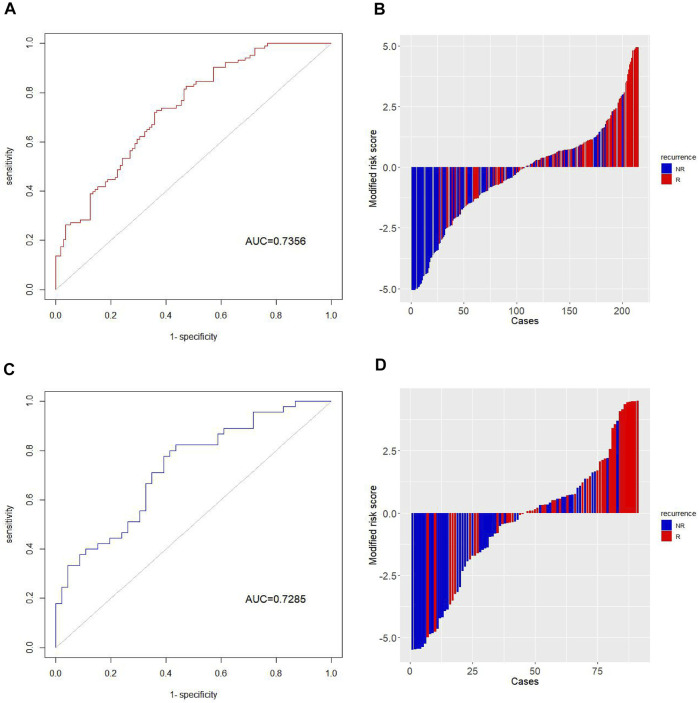
Performance evaluation of the risk assessment model.The ROC curves demonstrate the diagnostic performance of the model in distinguishing recurrent patients in the **(A)** training cohort and **(C)** validation cohort. The histograms show the risk score distribution in the **(B)** training and **(D)** validation cohorts. For convenience of display, the risk score is subtracted from the median and magnified 10 times to obtain the modified risk score.

In the training cohort, the performance of the risk assessment model was good, with an AUC value of 0.7356 (Figures 5A, B). Subsequently, we assessed the robustness and accuracy of this 18-mRNA signature by applying the same statistical model to the validation cohort. In the validation cohort, the 18-mRNA biomarkers also showed significant diagnostic accuracy in identifying postoperative recurrence in patients with liver cancer, with an AUC value of 0.7285 (Figures 5C, D).

Liver cancer is multi-centric and is significantly affected by background diseases. Different disease backgrounds may have influenced the results of the model. Therefore, we tried to verify whether the 18-mRNA signature can show good predictive performance in different etiological sources of liver cancer subgroups. Restricted by limited etiological data on patients, we could only divide the samples into ALD (n = 57) and HVI (n = 98) subgroups. Within the ALD subgroup, we randomly re-divided the training and validation cohorts in a 7:3 ratio and reconstructed a logistic model with the 18 mRNAs based on the training cohort to predict recurrence in patients in the ALD group and validated them in the validation cohort. The same procedure was performed for the HVI subgroup. These were executed to reduce the effect of background disease on the prediction results of the 18 mRNAs. In both subgroups, the 18-mRNA logistic regression models exhibited good and similar predictive performances. In the ALD subgroup, the AUC values of the 18-mRNA model were 0.8107 and 0.7273 on the training and validation sets, respectively. In the HVI subgroup, the AUC values were 0.8795 and 0.7764 for the training and validation sets, respectively (Supplementary Figure S3A–D). In summary, the 18-mRNA signature performed well in predicting liver cancer of different etiological sources (viral infection and alcohol-related), suggesting that our predictive markers may be universally applicable for predicting the recurrence of liver cancer from different etiological sources.

In order to form a comparison with the logistic regression model constructed by the above method, we used two other variable screening methods and constructed three different logistic models: a logistic regression model without variable filtering (model 2), a logistic regression model with variable filtering by stepwise regression (model 3), and a logistic regression model with variable filtering by L1 regularization (model 4). The prediction results of these three models are good in the training set, but the results in the validation set are much worse than those in the training set (Supplementary Figure S4A–F), that is, the models constructed by the three methods show serious overfitting. In contrast, the logistic model constructed after filtering with the random forest algorithm showed similar results and good predictions for both the training and validation cohorts.

We also compared the risk score with the StromalScore, ImmuneScore, and ESTIMATEScore. The StromalScore, ImmuneScore, and ESTIMATEScore were calculated based on the expressions of mRNAs. In the training set, the AUC values of the StromalScore, ImmuneScore, and ESTIMATEScore for predicting recurrence of liver cancer were 0.5581, 0.5599, and 0.5643, respectively, and 0.5923, 0.5942 and 0.5948 in the validation set, respectively (Supplementary Figure S5A–F). This indicated that the risk score proposed in our study had better performance in predicting the recurrence of liver cancer compared to StromalScore, ImmuneScore, and ESTIMATEScore.

### Correlation Between Risk Score and Immune Cells

**FIGURE 6:**
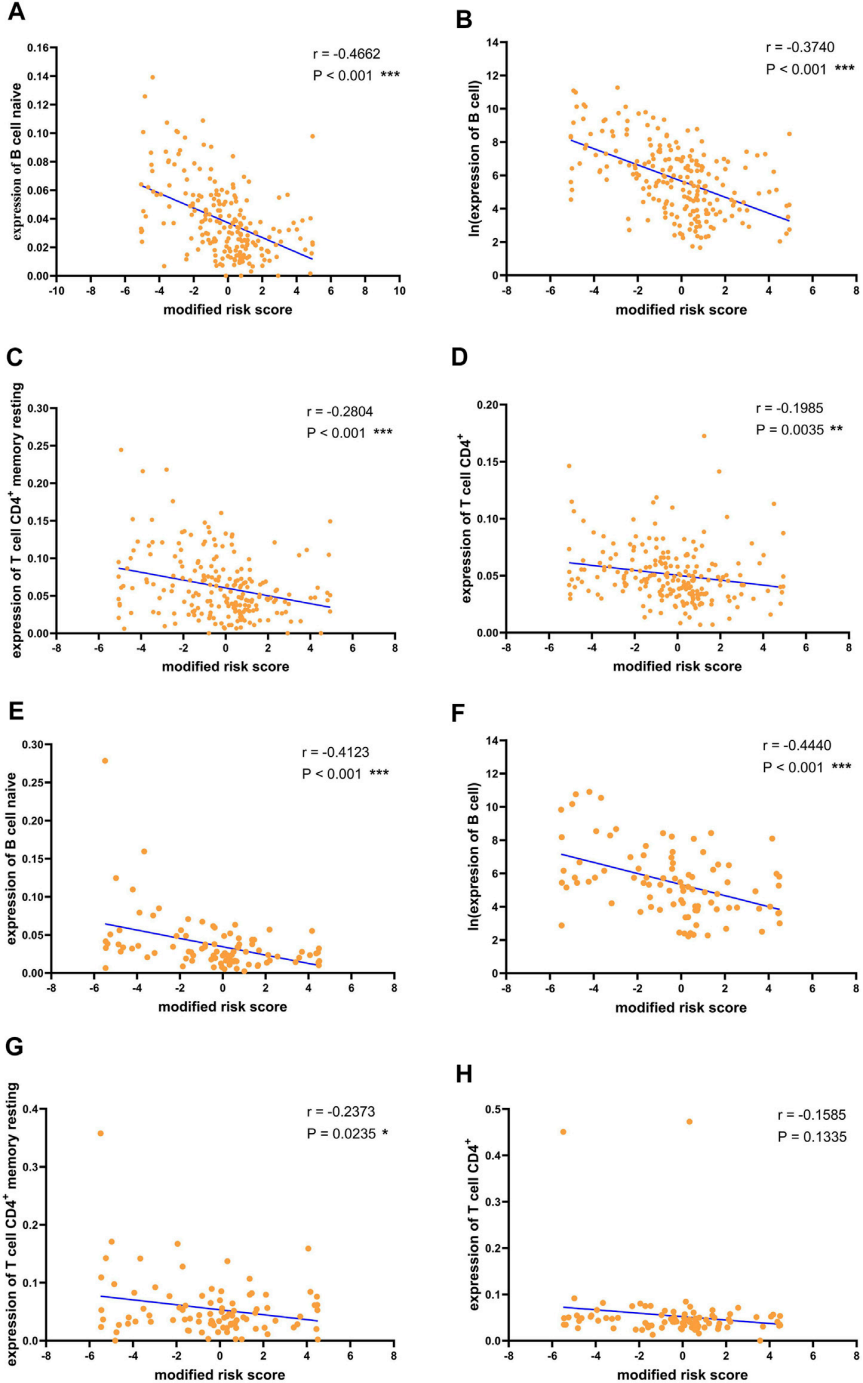
Correlation between risk scores and immune cells.Correlation between risk score and **(A)** B cell naive **(B)** B cell **(C)** T cell CD4^+^ memory resting **(D)** T cell CD4^+^ in the training data. Correlation between risk score and **(E)** B cell naive **(F)** B cell **(G)** T cell CD4^+^ memory resting **(H)** T cell CD4^+^ in the validation data. For convenience of display, the risk score is subtracted from the median and magnified 10 times to obtain the modified risk score. *p < 0.05, **p < 0.01 and ***p < 0.001.

The previous analysis results showed that the recurrence of liver cancer after surgery was significantly associated with the expression of immune cells. Furthermore, to confirm the relationship between the risk of recurrence and immune infiltration, we performed correlation analysis between the recurrence risk score estimated using the previously established risk assessment model and immune cells. In the training cohort, B cell naive, B cell, T cell CD4^+^ memory resting, and T cell CD4^+^ expression levels were significantly negatively correlated with the risk score (Figures 6A–D). The negative association between these immune cells and the risk score was also verified in the validation cohort (Figures 6E–H). The results demonstrated the validity of the risk score predicted by the 18-mRNA model and further confirmed the negative association between these four immune cells and postoperative recurrence of liver cancer.

### Prognostic Analysis Using the Risk Scores in the Training and Validation Sets

**FIGURE 7:**
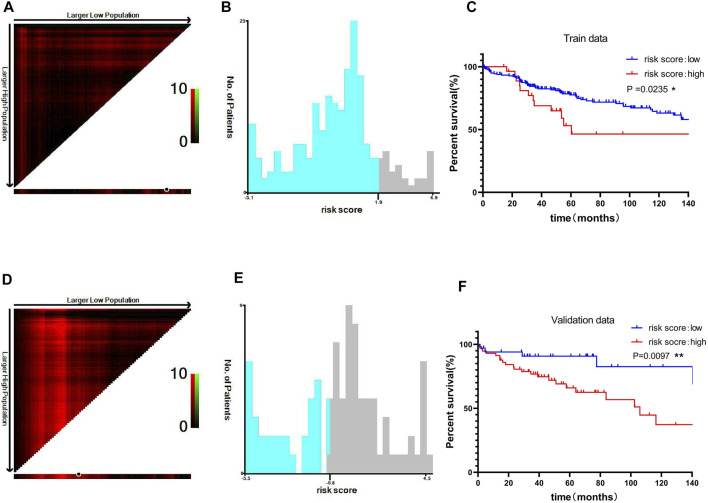
Prognostic analysis using the risk scores in the training and validation sets. Optimal cutoff value of the risk score divided by X-tile software in the training data **(A, B) (C)** Kaplan-Meier curve of the two groups divided by the cutoff value in the training data. The optimal cutoff value of the risk score was divided by the X-tile software in the validation data **(D, E) (F)** Kaplan-Meier curve of the two groups divided by the cutoff value in the validation data. *p < 0.05, **p < 0.01 and ***p < 0.001.

To improve the clinical prognostic significance of the risk score, we then grouped patients by risk score and performed Kaplan-Meier survival analysis. X-tile software was used to obtain the best truncation value, and patients in the training cohort were divided into low-risk and high-risk groups (Figures 7A, B). We observed a significant difference in overall survival (OS) between the low-risk and high-risk groups in the training cohort (*p* = 0.0235) (Figure 7C). Similarly, in the validation cohort, there was a significant difference in the prognosis between the high- and low-risk groups classified by the X-tile software (*p* = 0.0097) (Figures 7D–F). The results indicated that risk score had good prognostic value for liver cancer patients.

### Univariate and Multivariate Analyses of the mRNA Signature Prognostic Abilities

To verify the prognostic value of the 18-mRNA signature independently from the clinicopathological characteristics, we performed Cox univariate and multivariate analyses that included 18-mRNA risk score, age, sex, histologic grade, and TNM stage as co-variables in the training and validation cohorts. In univariate and multivariate analyses of the training cohort, TMN stage was found to be related to OS (Supplementary Figure S6A–B). However, in univariate and multivariate analyses of the validation cohort, the TMN stage was no longer significantly correlated with OS, and the risk score was a significant variable related to OS (Supplementary Figure S6C–D). HR values of the risk score were significant but not high, which was not perfect in clinical prognosis prediction. In addition, histologic grade and TMN stage appeared to have high but not significant HR values, which may be due to excessive standard errors of the variables. (Katz and Hauck, 1993).

## Discussion

In this study, we constructed a risk scoring model with 18 mRNAs to predict post-hepatectomy recurrence of liver cancer. The 18 mRNAs were LOC644135, ELN, GULP1, ENSG00000248635, GPM6A, PI15, SGPP2, TMEM200C, CDH3, SELP, CCBE1, IGLV1-44, IGLV2-11, IGLL5, IGHV3-23, IGHV5-51, IGHG2, and CD79A.The risk assessment model could accurately distinguish between low- and high-risk samples for the recurrence of liver cancer after resection, with good prognostic performance. The identified 18 mRNAs also had good predictive performance in liver cancer samples caused by different etiologies. B cell naive, B cell, T cell CD4^+^ memory resting, and T cell CD4^+^ were significantly different in recurrence versus non-recurrence liver cancer samples and were found to be negatively correlated with the risk scores predicted by the constructed model by correlation analysis.

Compared to the classification algorithms in machine learning, regression analysis has better explanatory power for variables. The advantage of our method is that it combines random forest with regression analysis to screen biomarkers of liver cancer recurrence and construct a formulaic risk assessment model of liver cancer recurrence, which ensures the interpretability of variables with good predictive performance. We also constructed a logistic regression model without feature screening, a stepwise regression logistic model, and an L1 regularized logistic model, which revealed that the random forest-based logistic regression model had better generalization performance on the validation set. Meanwhile, compared to StromalScore, ImmuneScore, and ESTIMATEScore, our risk score showed better performance to predict recurrence risk of liver cancer.

In previous studies, machine learning methods have also been used to predict liver cancer recurrence. Wang et al.(Wang et al., 2020) used lasso and Cox regressions to screen five mRNAs to predict HCC recurrence, and the predicted AUC values for 1-year, 2-year, and 3-year RFS rates from the independent validation data were 0.752, 0.651, and 0.677, respectively. Iizuka et al.(Iizuka et al., 2003) used Fisher’s linear classifier algorithm to predict intrahepatic recurrence in hepatocellular carcinoma patients within 1 year after resection, using 18 mRNAs. In the validation sample, the predictive accuracy was 92.6% (25/27). Based on this, Somura et al.(Somura et al., 2008) selected three of the mRNAs and constructed a prediction model with the same algorithm, with a correct prediction accuracy of 81.4% (35/43) in the validation set. However, selection bias or publication bias in the small sample sizes may produce inflated over-promising results. (Ntzani and Ioannidis, 2003). In addition, a few previous studies have used a large number of genetic biomarkers to predict the prognosis of liver cancer. (Lee et al., 2006; Hoshida et al., 2008; Woo et al., 2008). However, due to the different sample selection criteria and definition of the outcomes, there was little gene overlap between these studies. To our knowledge, the present study includes the largest sample size through the machine learning method compared to other studies in HCC recurrence prediction, which may enhance the validity of the gene signature predicted by our study. Our screened 18 mRNAs showed good performance in predicting liver cancer recurrence, with AUCs of 0.7356 and 0.7285 in the training and validation cohorts, respectively.

We report for the first time that LOC644135, ELN, GULP1, ENSG00000248635, GPM6A, and PI15 are associated with the risk of liver cancer recurrence. In addition, some genes that we identified have been reported to play a role in the development, recurrence, and metastasis of cancer in previous studies. It has been reported that Nudix hydrolase 21 promotes tumor growth and metastasis through modulating SGPP2 in gastric cancer. (Zhu et al., 2021). TMEM200C is reported to be hypomethylated, and candidate oncogenes linked to early metastasis in uveal melanoma. (Ness et al., 2021). CDH3, a classical cell adhesion molecule, has been reported to be related to a variety of human cancers. L. Li et al. (Li et al., 2019) found that Kruppel-like factor 4 (KLF4)-mediated upregulation of CDH3 inhibits the growth and migration of human hepatoma cells through GSK-3 β signaling. Overexpression of CDH3 has been reported to promote the movement of pancreatic cancer cells by interacting with P120ctn and activating Rho family GTPases. (Taniuchi et al., 2005). CDH3 has also been reported to be associated with prognosis in patients with colorectal adenocarcinoma and lung cancer. (Xu et al., 2019; Hsiao et al., 2020). SELP is a member of the selectin family of cell adhesion molecules, and it mediates heterotypic aggregation of activated platelets to cancer cells and adhesion of cancer cells to stimulated endothelial cells. (Chen and Geng, 2006). It has been demonstrated that SELP plays an important role in the growth and metastasis of human colon carcinoma *in vivo*. (Kim et al., 1998). CCBE1 is essential in lymphatic vascular development. Song et al.(Song et al., 2020) demonstrated the procarcinogenic role of CCBE1 in promoting lymphangiogenesis and metastasis in colorectal cancer, and it has been reported that targeting of CCBE1 by miR-330-3p promotes tumor metastasis in breast cancer. (Mesci et al., 2017). Our results contribute to further understanding of the impact of these genes on recurrence of liver cancer. It is interesting to note that many of the genes we report are immunoglobulin genes (IGLV1-44, IGLV2-11, IGLL5, IGHV3-23, IGHV5-51, IGHG2). Previous studies have shown that some immunoglobulins regulate the tumor microenvironment and influence the prognosis of patients with breast cancer, diffuse large B-cell lymphoma, and chronic lymphocytic leukemia (Baliakas et al., 2015; Zhang et al., 2015; Stamatopoulos et al., 2018; Xu-Monette et al., 2019), and our results suggest that the above immunoglobulin genes may play an important role in recurrence of liver cancer. CD79A is also an immune function-related gene. CD79A and CD79b molecule (CD79B) heterodimers are important signaling components of the B cell receptor (BCR) complex, which plays a crucial role in B cell development and antibody production. (Li et al., 2017b). These immune-related genes identified in our study may provide insights into the underlying mechanism of liver cancer recurrence.

Previous studies have reported that immune cells play a role in cancer recurrence. (Bindea et al., 2013; Chevalier et al., 2017; Zhou et al., 2019). The enrichment results showed that DE-mRNAs were enriched in many immune-related pathways. Therefore, on the basis of predicting liver cancer recurrence, we analyzed the differences in immune cells between patients with and without recurrence, and explored the relationship between the predicted risk of recurrence and differential immune cells in patients with liver cancer. We demonstrated that compared with the non-recurrence group, B cell naive, B cell, T cell CD4^+^ memory resting, and T cell CD4^+^ were significantly downregulated in the recurrence group and were inversely associated with the recurrence risk evaluated by the model we built. Previous studies have shown that tumor metastasis is associated with the presence of CD4^+^ T cells and B cells. Olkhanud et al. (Olkhanud et al., 2011) found that tumor-evoked regulatory B cells promote breast cancer metastasis by converting resting CD4^+^ T cells to T-regulatory cells. Ou et al.(Ou et al., 2015) found that tumor microenvironment B cells increase bladder cancer metastasis via modulation of the IL-8/androgen receptor (AR)/MMPs signals. In addition, Guy et al. (Guy et al., 2016) found that tumor-specific CD4^+^ T cells and B cells play important functions and major roles in anticancer immunity. Many studies have shown that a variety of immunosuppressive signals regulate the tumor microenvironment, which plays an important regulatory role in the process of tumorigenesis, and its heterogeneity can lead to multiple aspects, including patient prognosis and treatment response. (Fridman et al., 2012; Galon et al., 2014; Hui and Chen, 2015). Sun et al. (Sun et al., 2021) reported that recurrent HCC has a unique immune ecosystem compared with the primary HCC tumor microenvironment. Therefore, our findings may provide a new approach for the immunotherapy of liver cancer recurrence.

In conclusion, the risk assessment model developed in this study may serve as a complementary tool to provide useful information for predicting disease outcomes after hepatectomy in patients with liver cancer and guide adjuvant therapy. Meanwhile, the immune cells reported in this study could be the targets of immunotherapy for patients with liver cancer. While these predictions are valuable, the current study has some limitations. The 18-mRNA biomarkers were screened by bioinformatics and machine learning methods, and their expression and specific functions need to be validated by biological experiments. In addition, the predictive performance of the 18-mRNA risk assessment model constructed in our study needs to be validated using large independent samples.

## Data Availability

The original contributions presented in the study are included in the article/Supplementary Material, further inquiries can be directed to the corresponding authors.
