# Supplementary material for: Recurrence Risk of Liver Cancer Post-hepatectomy Using Machine Learning and Study of Correlation With Immune Infiltration
Source: Front Genet. 2021 Dec 8;12:733654. doi: 10.3389/fgene.2021.733654 (PMC8692778; doi:10.3389/fgene.2021.733654)
Supplement: Supplementary file 1 [file Image5.PDF]

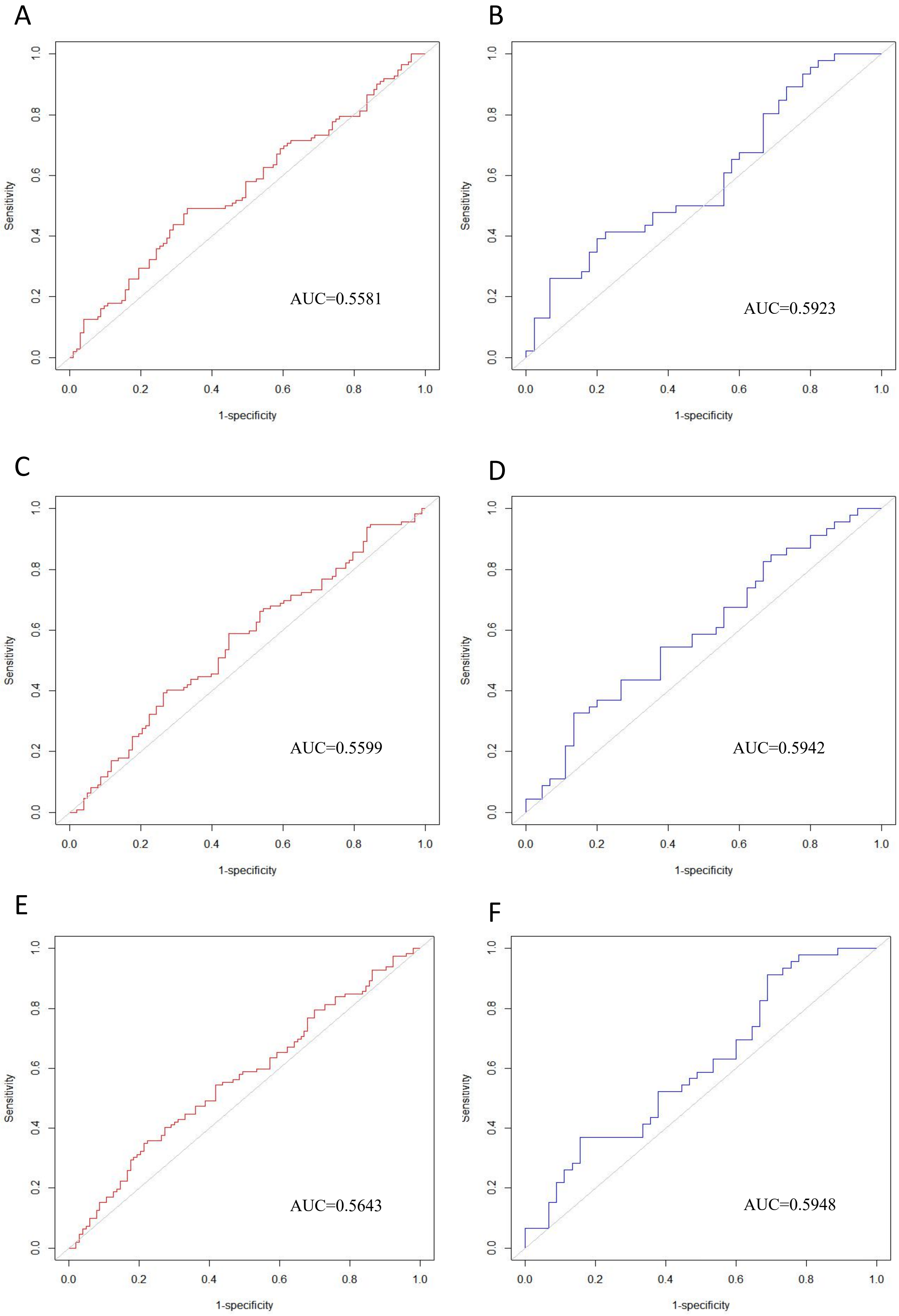

Supplementary Figure 5. Predictive performance of StromalScore, ImmuneScore, and ESTIMATEScore. ROC curves of StromalScore in the train set(A) and validation set (B). ROC curves of ImmuneScore in the train set(C) and validation set (D) . ROC curve of ESTIMATEScore in the train set(E) and validation set (F).
