# Supplementary material for: Recurrence Risk of Liver Cancer Post-hepatectomy Using Machine Learning and Study of Correlation With Immune Infiltration
Source: Front Genet. 2021 Dec 8;12:733654. doi: 10.3389/fgene.2021.733654 (PMC8692778; doi:10.3389/fgene.2021.733654)
Supplement: Supplementary file 2 [file Table1.PDF]

Supplementary Table 1. Clinical and pathological characteristics in different data sets

Characteristics	All(n=306)	Training data(n=215)	Validation data(n=91)
Age at diagnosis, years			
<60	143 (46.7%)	95 (44.2%)	48 (52.7%)
≥60	163 (63.3%)	120 (55.8%)	43 (47.3%)
Gender			
Female	101 (33.0%)	77 (35.8%)	24 (26.4%)
Male	205 (67.0%)	138 (64.2%)	67 (73.6%)
Histologic grade			
G1	45 (14.7%)	33 (15.3%)	12 (13.2%)
G2	142 (46.4%)	99 (46.0%)	43 (47.3%)
G3	105 (34.3%)	77 (35.8%)	28 (30.8%)
G4	10 (3.3%)	5 (2.3%)	5 (5.5%)
NA	4 (1.3%)	1 (0.5%)	3 (3.3%)
TMN stage			
I	144 (47.1%)	103 (47.9%)	41 (45.1%)
II	75 (24.5%)	49 (22.8%)	26 (28.6%)
III	66 (21.6%)	44 (20.5%)	22 (24.2%)
IV	2 (0.7%)	1 (0.5%)	1 (1.1%)
NA	19 (6.2%)	18 (8.4%)	1 (1.1%)
Recurrence			
Yes	148 (51.6%)	103 (47.9%)	45 (49.5%)
No	158 (48.4%)	112 (52.1%)	46 (50.5%)
