# Supplementary material for: Recurrence Risk of Liver Cancer Post-hepatectomy Using Machine Learning and Study of Correlation With Immune Infiltration
Source: Front Genet. 2021 Dec 8;12:733654. doi: 10.3389/fgene.2021.733654 (PMC8692778; doi:10.3389/fgene.2021.733654)
Supplement: Supplementary file 3 [file Table2.PDF]

Supplementary Table 2. Clinical and pathological characteristics in recurrence and non-recurrence groups

Characteristic	NA	category	All (n=306)	Non-recurrence (n=158)	Recurrence (n=148)	P value
Age , median[IQR]	0		61.0[52.0,68.0]	61.0[52.0,68.0]	61.0[52.0,68.0]	0.745
Gender , n(%)	0	0	101(33.007)	52(32.911)	49(33.108)	0.971
		1	205(66.993)	106(67.089)	99(66.892)	
Grade , n(%)	4	1	45(14.901)	26(16.561)	19(13.103)	0.535
		2	142(47.020)	76(48.408)	66(45.517)	
		3	105(34.768)	49(31.210)	56(38.621)	
		4	10(3.311)	6(3.822)	4(2.759)	
TMN stage , n(%)	19	1	144(50.174)	92(61.745)	52(37.681)	<0.001
		2	75(26.132)	38(25.503)	37(26.812)	
		3-4	68(23.693)	19(12.752)	49(35.507)	
