# Supplementary material for: Recurrence Risk of Liver Cancer Post-hepatectomy Using Machine Learning and Study of Correlation With Immune Infiltration
Source: Front Genet. 2021 Dec 8;12:733654. doi: 10.3389/fgene.2021.733654 (PMC8692778; doi:10.3389/fgene.2021.733654)
Supplement: Supplementary file 4 [file Image6.PDF]

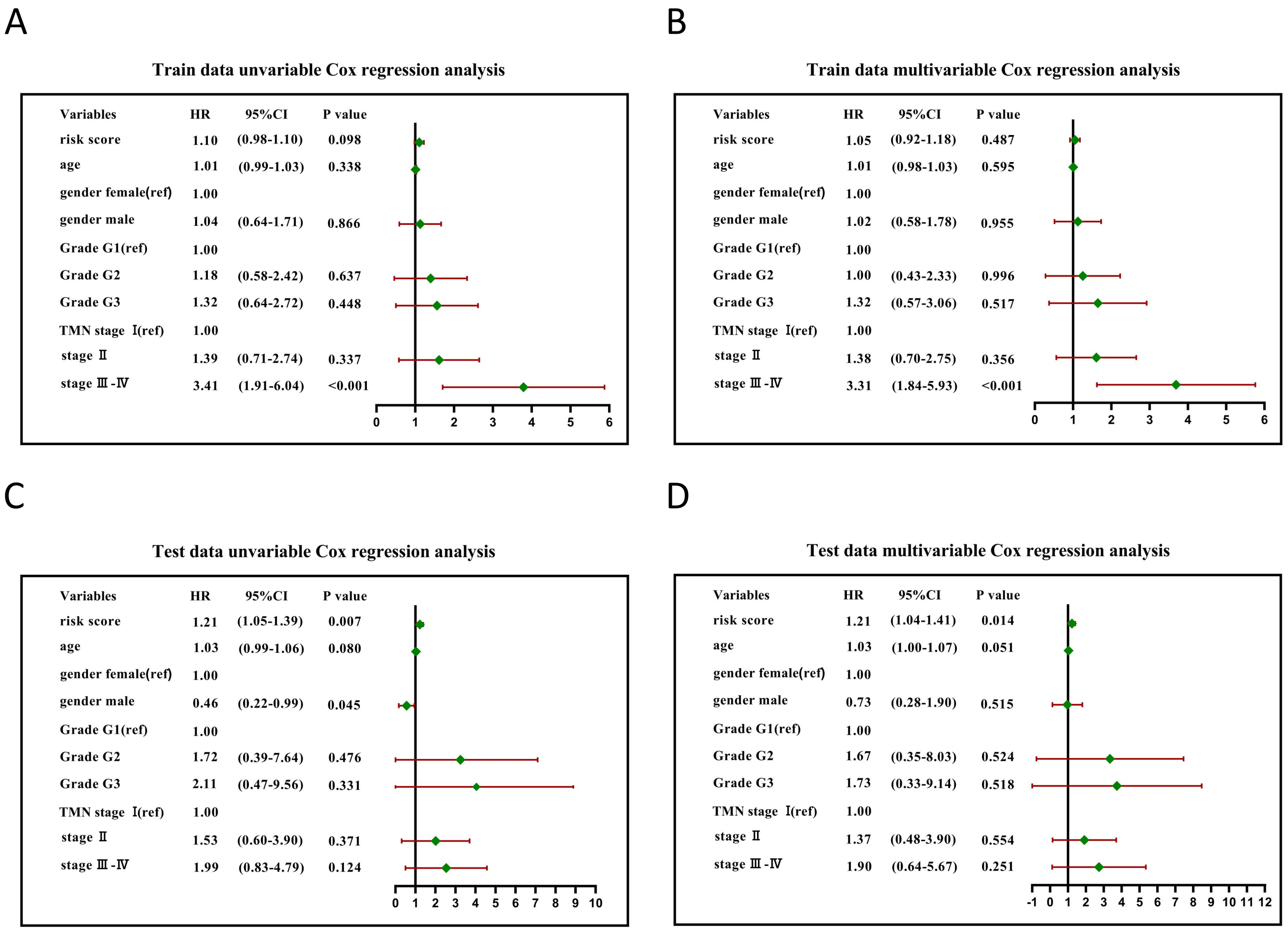

Supplementary Figure 6. Forest plot summary of analyses of overall survival. Univariate and multivariate analyses of the risk score, age, sex, histological grade, and TNM stage in training data(A, B) and validation data(C, D). The green points on the transverse lines represent the hazard ratio (HR), and the red transverse lines represent 95% CIs. Risk score and age are continuous variables, and gender, histological grade, and TNM stage are discontinuous variables.
