# Supplementary material for: Recurrence Risk of Liver Cancer Post-hepatectomy Using Machine Learning and Study of Correlation With Immune Infiltration
Source: Front Genet. 2021 Dec 8;12:733654. doi: 10.3389/fgene.2021.733654 (PMC8692778; doi:10.3389/fgene.2021.733654)
Supplement: Supplementary file 5 [file Image4.PDF]

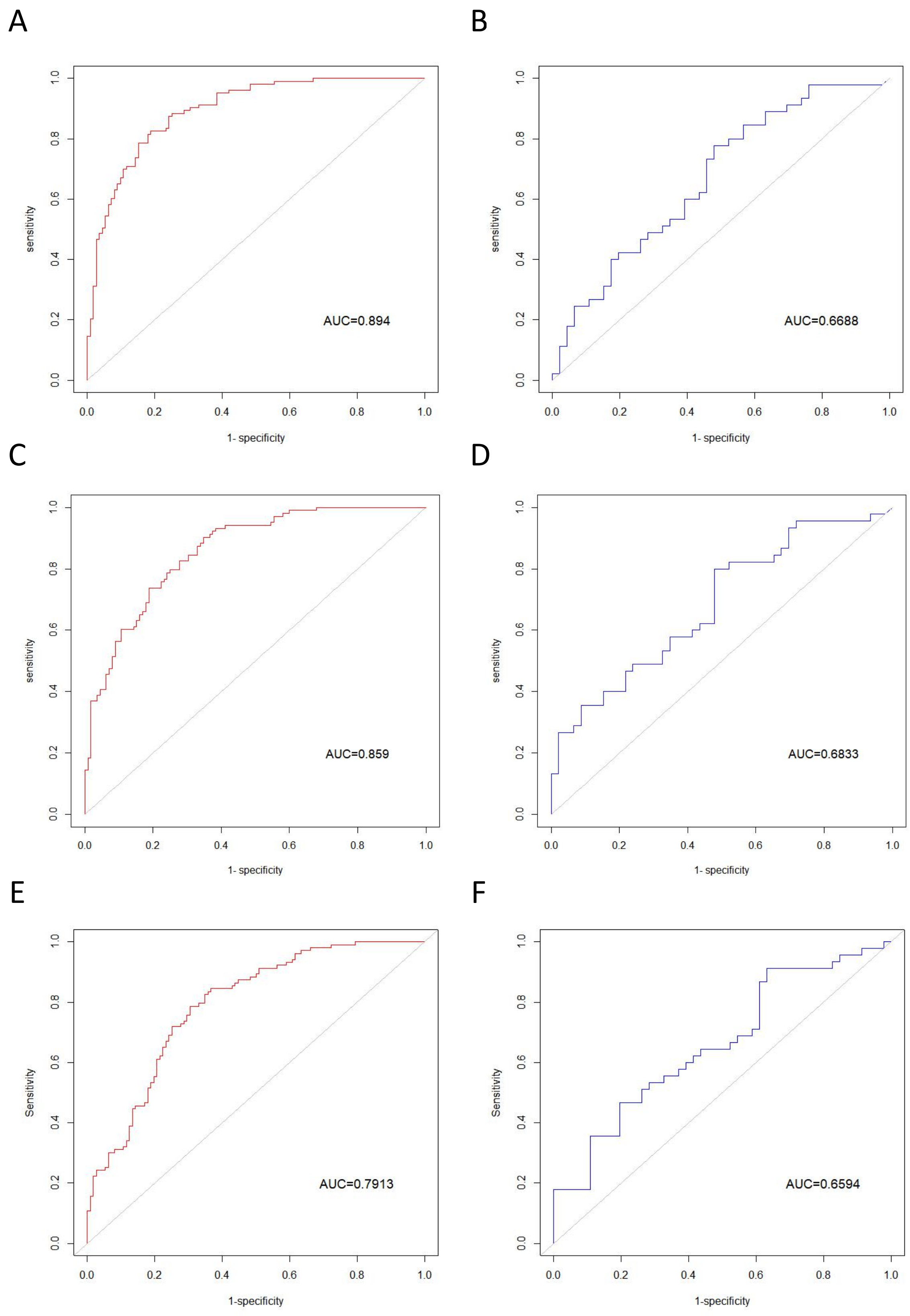

Supplementary Figure 4. Predictive performance of three logistic regression models.(A) ROC curve of model 2 on the training cohort. (B) ROC curve of model 2 on the validation cohort. (C) ROC curve of model 3 on the training cohort. (D) ROC curve of model 3 on the validation cohort. (E) ROC curve of model 4 on the training cohort. (F) ROC curve of model 4 on the validation cohort.
