# Supplementary material for: Recurrence Risk of Liver Cancer Post-hepatectomy Using Machine Learning and Study of Correlation With Immune Infiltration
Source: Front Genet. 2021 Dec 8;12:733654. doi: 10.3389/fgene.2021.733654 (PMC8692778; doi:10.3389/fgene.2021.733654)
Supplement: Supplementary file 6 [file Image2.PDF]

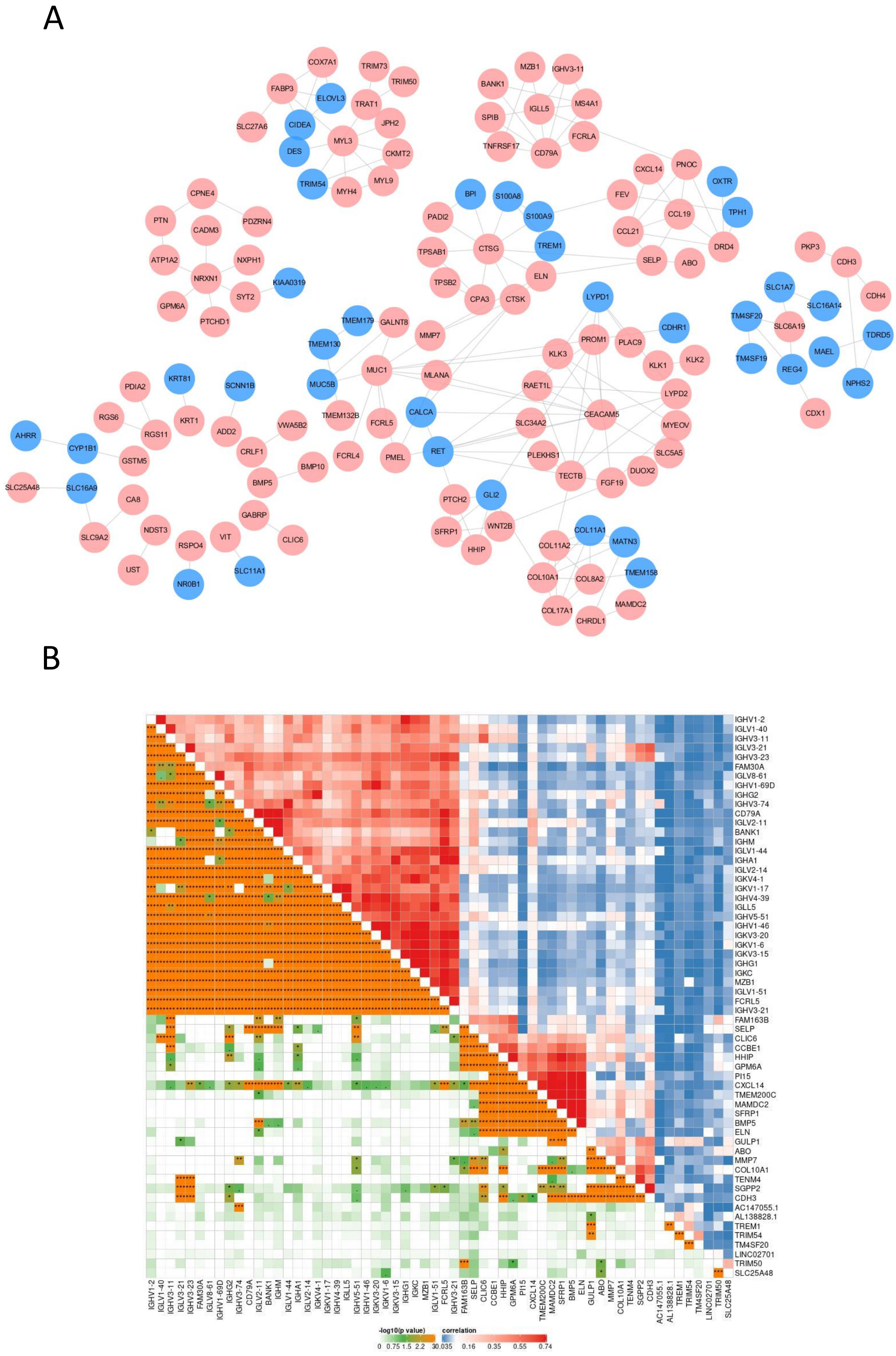

Supplementary Figure 2. The link between DE-mRNAs.(A) Protein-protein interaction networks of all DE-mRNAs. Pink: down-regulated in the recurrence group; Blue: up-regulated in the recurrence group. (B) Correlation between overlapping DE-mRNAs.
