# Supplementary material for: Recurrence Risk of Liver Cancer Post-hepatectomy Using Machine Learning and Study of Correlation With Immune Infiltration
Source: Front Genet. 2021 Dec 8;12:733654. doi: 10.3389/fgene.2021.733654 (PMC8692778; doi:10.3389/fgene.2021.733654)
Supplement: Supplementary file 7 [file Image3.PDF]

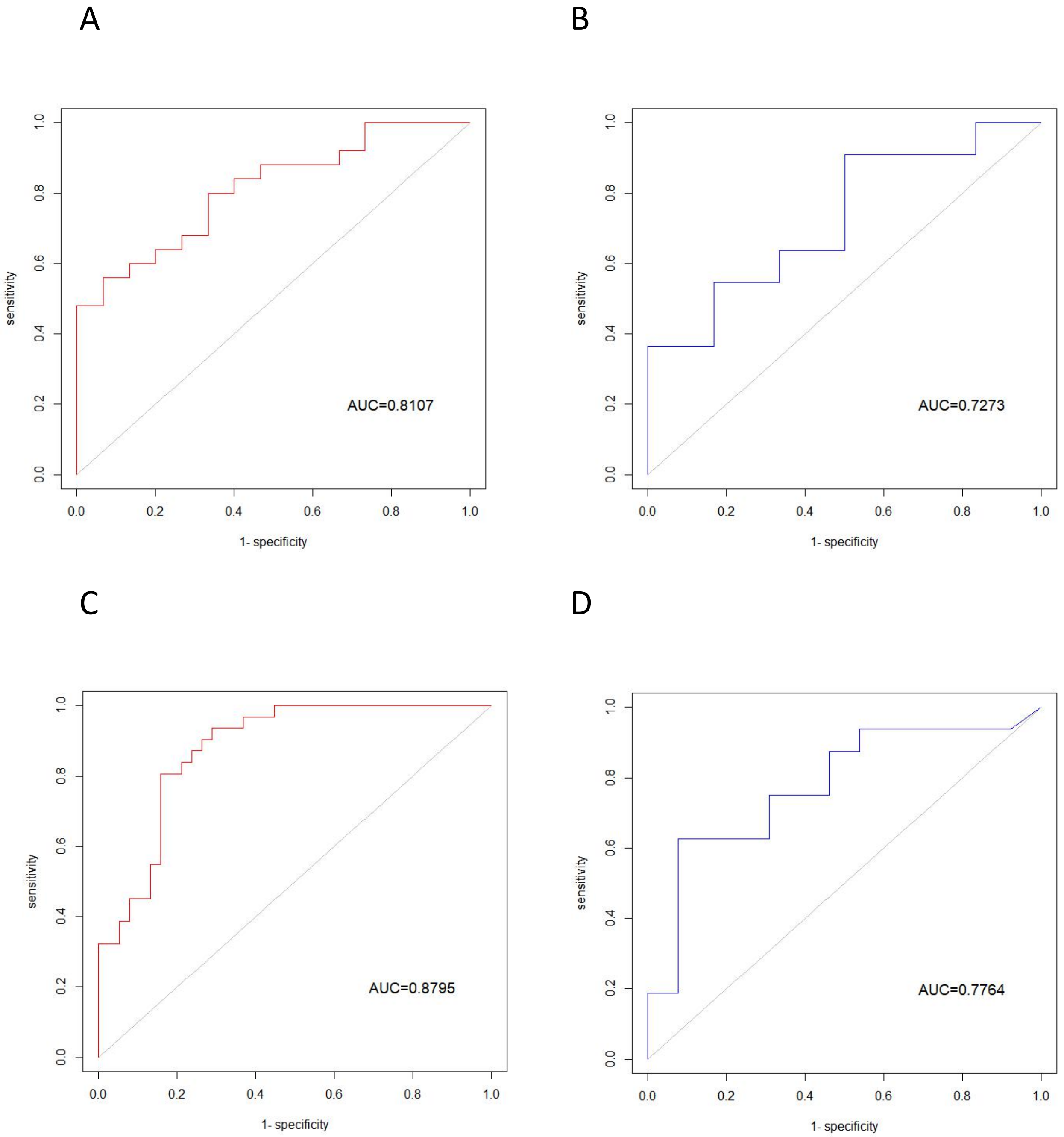

Supplementary Figure 3. Predictive performance of 18 mRNAs for recurrence of different subtypes of liver cancer.(A) ROC curve of alcohol-associated liver disease subgroup on the training cohort. (B) ROC curve of alcohol-associated liver disease subgroup on the validation cohort. (C) ROC curve of hepatitis virus infection associated subgroup on the training cohort. (D) ROC curve of hepatitis virus infection associated subgroup on the validation cohort.
