# Supplementary figures and images for: Recurrence Risk of Liver Cancer Post-hepatectomy Using Machine Learning and Study of Correlation With Immune Infiltration
Source: Front Genet. 2021 Dec 8;12:733654. doi: 10.3389/fgene.2021.733654 (PMC8692778; doi:10.3389/fgene.2021.733654)

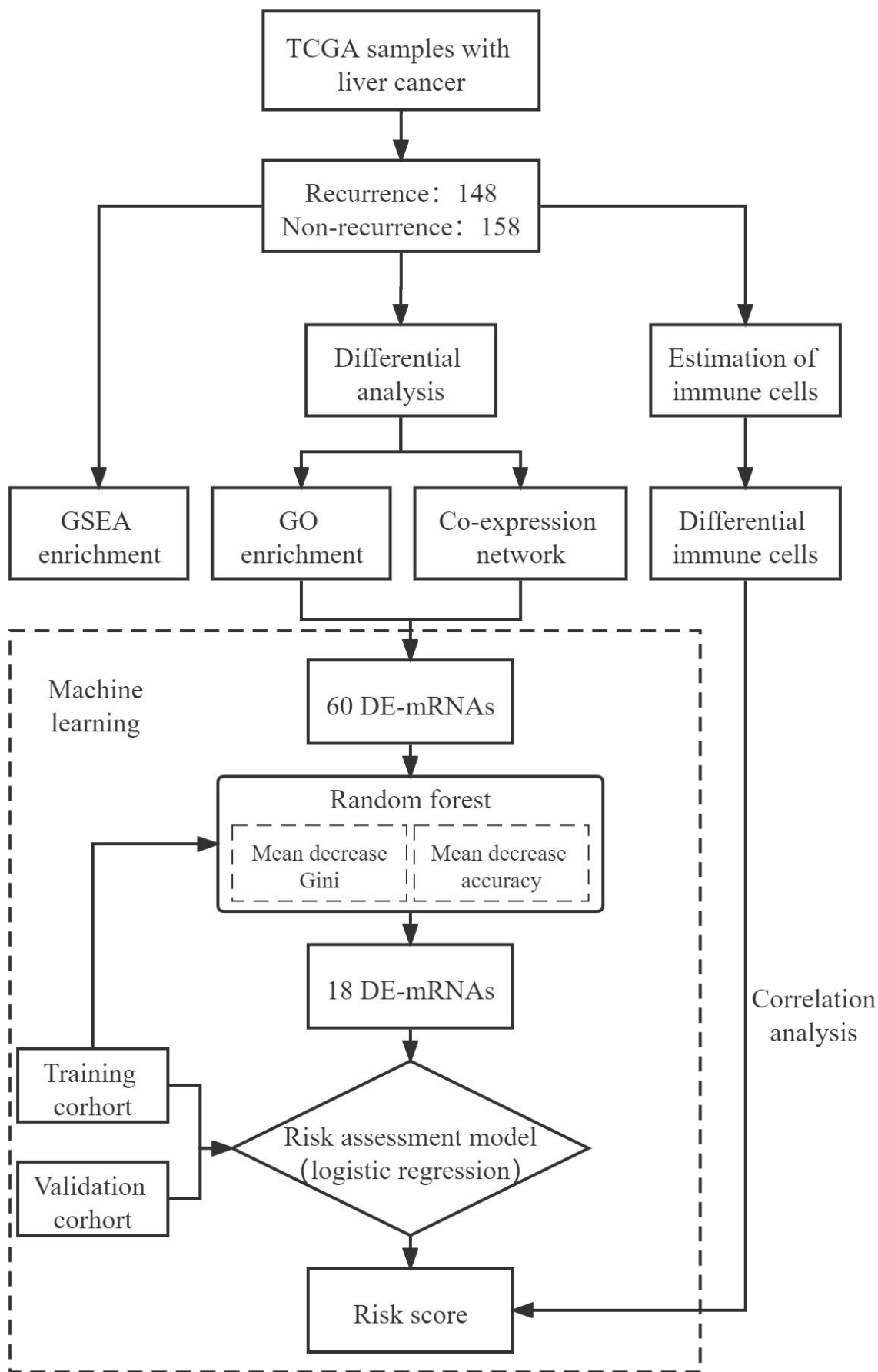

**Supplementary Figure 1. The overall flow chart of this study.**

Supplement: Supplementary file 8 [file Image1.PDF]
